# Age-independent increasing prevalence of Human Papillomavirus-driven oropharyngeal carcinomas in North-East Italy

**DOI:** 10.1038/s41598-020-66323-z

**Published:** 2020-06-09

**Authors:** Annarosa Del Mistro, Helena Frayle, Anna Menegaldo, Niccolò Favaretto, Silvia Gori, Piero Nicolai, Giacomo Spinato, Salvatore Romeo, Giancarlo Tirelli, Maria Cristina da Mosto, Jerry Polesel, Paolo Boscolo Rizzo

**Affiliations:** 1 Immunology and Diagnostic Molecular Oncology Unit, Veneto Institute of Oncology IOV – IRCCS, Via Gattamelata, 64, 35128 Padova, Italy; 2 Department of Neurosciences DNS, Section of Otolaryngology, University of Padova, P.le Ospedale 1, 31100 Treviso, Italy; 3 Department of Neurosciences DNS, Section of Otolaryngology, University of Padova, Via Giustiniani 2, 35128 Padova, Italy; 4 Department of Surgery, Oncology and Gastroenterology, Section of Oncology and Immunology, University of Padova, Via Gattamelata, 64, 35128 Padova, Italy; 5 Anatomical Pathology Unit, San Donà di Piave Hospital, Azienda ULSS 4 Veneto Orientale, Via Nazario Sauro 25, 30027 San Donà di Piave, VE Italy; 6 Head and Neck Department, Cattinara Hospital, University of Trieste, Strada di Fiume 447, 34149 Trieste, Italy; 7 Unit of Cancer Epidemiology, Aviano National Cancer Institute, IRCCS, Via Franco Gallini, 2, 33081 Aviano, PN Italy

**Keywords:** Medical research, Diagnostic markers, Prognostic markers, Cancer

## Abstract

HPV-driven oropharyngeal carcinomas (OPCs) show geographical variations with increasing temporal trends in several areas. We investigated their frequency and clinical outcomes within a prospective multicenter cohort study in North-East Italy. A tumor was defined as HPV-driven by using at least two different biomarkers, usually HPV-DNA positivity and p16^INK4A^ overexpression. Different survival outcomes were compared among patients with HPV-driven and non-HPV-driven tumors. Overall, 42/130 (32.3%) patients with newly diagnosed OPC during the period 2000–2018 resulted HPV-driven; HPV16 was involved in 37 cases (88%), HPV33 in 3 cases (7%), HPV58 and HPV18 in 1 case each. Over time, HPV-driven cases raised from 16.7% (6/36) during 2000–2006 to 46.1% (24/52) during 2013–2018 (p < 0.001). The increase in HPV-driven OPCs was more marked in females than males (p = 0.010), and the frequency of HPV-driven cases was similar in the different age groups. In comparison to cases with non-HPV-driven tumors, a significantly (p < 0.001) better progression-free and overall survival were recorded among patients affected by HPV-driven OPC. The prevalence of HPV-driven OPC cases has been significantly increasing during the last two decades also in North-East Italy and was associated with favorable outcome. OPCs driven by non-HPV16 oncogenic types were restricted to patients older than 68-yrs.

## Introduction

Oropharyngeal squamous cell carcinomas (OPC) are causally associated to chemical exposure (tobacco smoking and/or alcohol) or persistent infection by high-risk HPV (human papillomavirus) types^1,2^. In comparison to chemical-induced tumors, HPV-driven cancers represent a separate entity, characterized by a distinct molecular pathway, a better response to treatment and an improved prognosis^3^. In particular, patients with HPV-driven OPC show good response to chemotherapy and radiotherapy with a good control of loco-regional relapse, but not of distant metastases. Among the factors influencing the prognosis, stage and smoking habit are well recognized^4,5^, while studies on the search for biomarkers able to predict patients’ risk of relapse are ongoing^6^.

Large variations on the frequency of HPV-driven OPC in different geographic areas exist, with highest (50–60%) figures in United States, Canada and Northern Europe, and lower in Southern Europe including Italy; in this latter the frequency is around 25–30%^7^. In particular, within Europe the frequency was reported to range from 24.2% to 56.5% in Southern and Northern countries, respectively^8^. Data on HPV prevalence in OPC cases showing differences among Italian regions have also been reported, with figures ranging from 11% to 75%^9–12^; small groups of patients were included and different HPV detection methods were used.

In most areas the incidence of HPV-driven OPC is on the rise, a trend more pronounced in the countries with higher frequencies^2,13,14^.

The improved prognosis of HPV-driven OPC has prompted investigations on aspects such as the best strategy to correctly distinguish HPV-driven from non-HPV-driven cases^15^, predictive biomarkers of relapse^5^, and possible modifications of the therapeutic regimens to reduce correlated morbidity^16^.

The present study reports a time-trend analysis of HPV-driven cases over roughly two decades and analyzes the clinical outcome in relation to viral and cellular parameters.

## Results

A total of 130 patients with primary OPC (median age: 65 years; range 41–85 years), were included in the study; they were predominantly males, no peak age at diagnosis was observed, and the tonsil was the most frequently involved sub-site. Anatomo-clinical data are summarized in Table 1.

**Table 1:** Distribution of 130 patients with oropharyngeal carcinomas according to socio-demographic and clinical characteristics.

	Total patients	HPV-driven cancers		CMH test^a^
	n	n	(%)	
**Gender**				
Female	35	18	(51.4)	
Male	95	24	(25.3)	p = 0.005
**Age (years)**				
<65	68	21	(30.9)	
≥65	62	21	(33.9)	p = 0.717
**Year of diagnosis**				
2000–2006	36	6	(16.7)	
2007–2012	42	12	(28.6)	
2013–2018	52	24	(46.2)	p < 0.001
**Cancer subsite**				
Base of tongue	28	11	(39.3)	
Tonsil	84	30	(35.7)	
Other	18	1	(5.6)	p = 0.021^c^
**TNM stage (7th ed.)**				
I-II	13	3	(23.1)	
III	23	11	(47.8)	
IV	94	28	(29.8)	p = 0.995
**TNM stage (8th ed.)**				
I-II	38	27	(71.1)	
III	27	15	(55.6)	
IV	65	0	(0.0)	p < 0.001
**Grading**				
Well differentiated	10	2	(20.0)	
Moderately differentiated	53	13	(24.5)	
Poorly differentiated	55	20	(36.4)	p = 0.143
*Unknown*	12	6	*(50.0)*	
**Tobacco smoking**				
Never	26	18	(69.2)	
Former	30	10	(33.3)	
Current	74	14	(18.9)	p < 0.001^c^
**Alcohol drinking**^**b**^				
Never	54	30	(55.6)	
Former	13	1	(7.7)	
Current	61	10	(16.4)	p < 0.001^c^

^a^Cochran-Mantel-Haenszel test; ^b^Two patients did not report drinking habits; ^c^Fisher exact test.

### Frequency and characteristics of HPV-driven tumors

Overall, 42/130 (32.3%) cases resulted HPV-driven (Table 1); prevalence was higher among never smokers and never drinkers (p < 0.001), and among women than among men (51.4% vs. 25.3%; p = 0.005) with a male/female prevalence ratio of 0.49 (95% CI: 0.21–0.69). Conversely, patients’ age at diagnosis was similar to that of non-HPV-driven cases.

**Figure 1:**
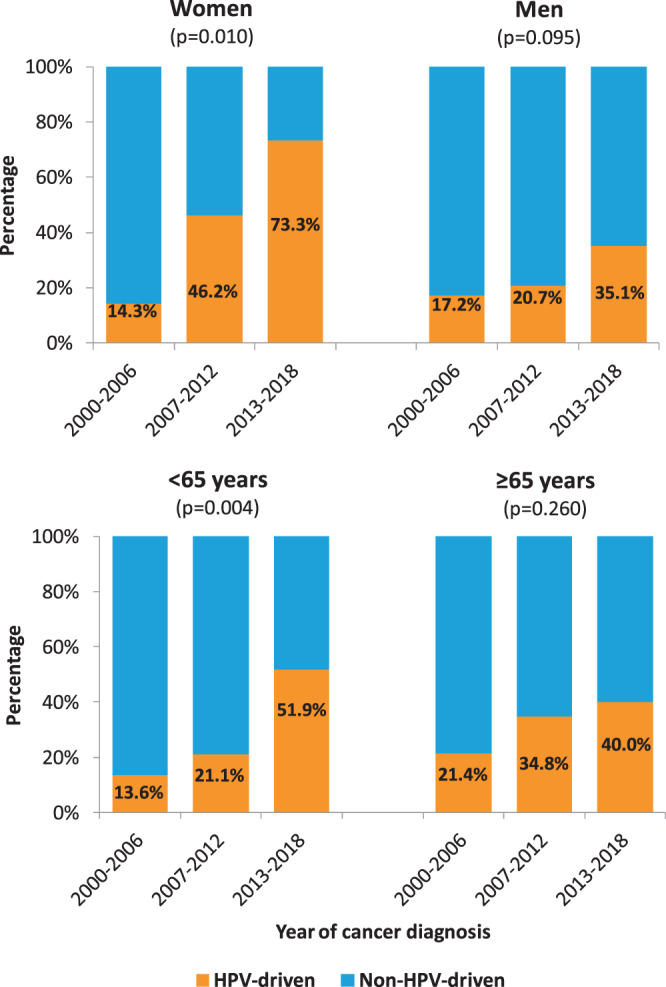
Prevalence of HPV-driven OPC by gender, age and year of diagnosis. OPC cases are grouped in three periods and the proportions of HPV-driven over total cases are shown.

**Figure 2:**
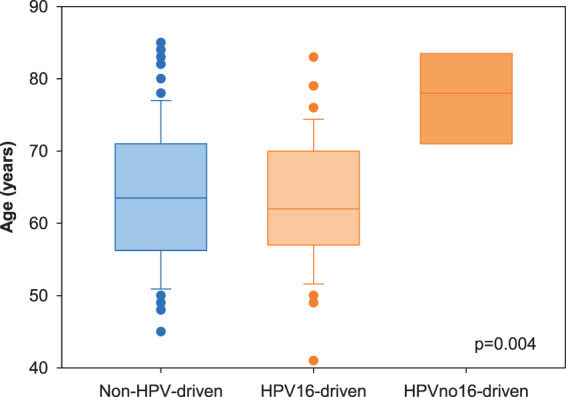
Box plot analysis of age at diagnosis of patients with non-HPV-driven and HPV-driven (HPV16 or other hrHPV types) OPC.

A statistically significant increase over time was recorded; HPV-driven cases raised from 16.7% (6/36) during 2000–2006 to 46.1% (24/52) during 2013–2018 (p < 0.001; Table 1). By stratifying for gender and median age, the increasing trend was evident in both genders, more pronounced and significant in female (p = 0.010) and in younger patients (p = 0.004; Fig. 1). HPV16 was involved in 37 cases (88%), HPV33 in 3 (7%) and HPV58 and HPV18 in 1 case each. HPV16 was more frequently detected among younger patients (median 62 years, range 41–83), whereas other types were identified only among older patients (median 78 years, range 69–84) (p = 0.004). A box plot of age for HPV-negative, HPV16-positive and HPVno16-positive cases is reported in Fig. 2. For HPV-driven cases, the differences between patients with HPV16-driven OPC and patients with other hrHPV types are summarized in Table 2.

**Table 2:** HPV-driven OPC cases: anatomo-clinical differences by viral type. HPVno16+ cases = HPV types 33 (3 cases), 18 and 58 (1 case each).

	HPV16 + N = 37	HPVno16+ N = 5	Fisher exact test
**Gender**			
Female	16	2	p = 1.000
Male	21	3	
M/F ratio	1,3	1,5	
**Age (years)**			
Median	62	78	p = 0.004^a^
Range	41–83	69–84	
**Year of diagnosis**			
2000–2006	6 (100%)	0 (0%)	p = 0.672
2007–2012	11 (92%)	1 (8%)	
2013–2018	20 (83%)	4 (17%)	
**Cancer subsite**			
Tonsil	28 (76%)	3 (60%)	p = 0.131
Base of tongue	9	2	
**TNM stage (8th ed.)**			
I-II	23 (62%)	4 (80%)	p = 0.639
III-IV	14	1	
**Tobacco smoking**			
Never	17 (46%)	1 (20%)	p = 0.501
**Alcohol drinking**			
Never	26 (70%)	4 (80%)	p = 1.000
**Clinical outcome**			
Complete remission	34 (92%)	5 (100%)	
OS status: 1	8	0	
PFS status: 1	9	0	
Exitus: 1	8	0	

^a^Wilcoxon test.

Viral load was analyzed by real-time PCR, and a valid result is available for 36/37 HPV16-driven tumors; a median of 123,5 HPV16 copies/cell (range 1,3–1375; >100 in 50%) was detected.

No correlations with socio-demographic and clinical characteristics emerged (Supplementary Table 1).

The HPV-driven status was determined by HPV-DNA positivity and p16 overexpression in 30 cases, HPV-DNA positivity and positivity for E6-mRNA and/or E6 oncoprotein and/or anti-E6/E7 antibodies in 5 cases, and HPV-DNA positivity and high HPV16 viral load (median 297,2 copies/cell) in 7 cases. HPV16 DNA was detected at low viral load (2 copies/cell) in one p16-negative, non-HPV-driven case.

The expression of p16 protein was evaluated in 66 cases (30 HPV-driven and 36 non-HPV-driven). Overexpression was recorded in all HPV-driven and in 5/36 (13,9%) non-HPV-driven tumors tested (Supplementary Table 2). The remaining 12 HPV-driven cases not analysed for p16 were mainly diagnosed in the 2000–2006 period, when p16 analysis was not routinely performed.

### Clinical outcome

Overall, by 31 July 2019 (median follow-up length: 33 months, Q1-Q3: 14–60 months) death occurred in 70/130 (53.8%) patients; 8/42 (19.0%) of HPV-driven and 62/88 (70.4%) of non-HPV-driven cases, respectively.

**Figure 3:**
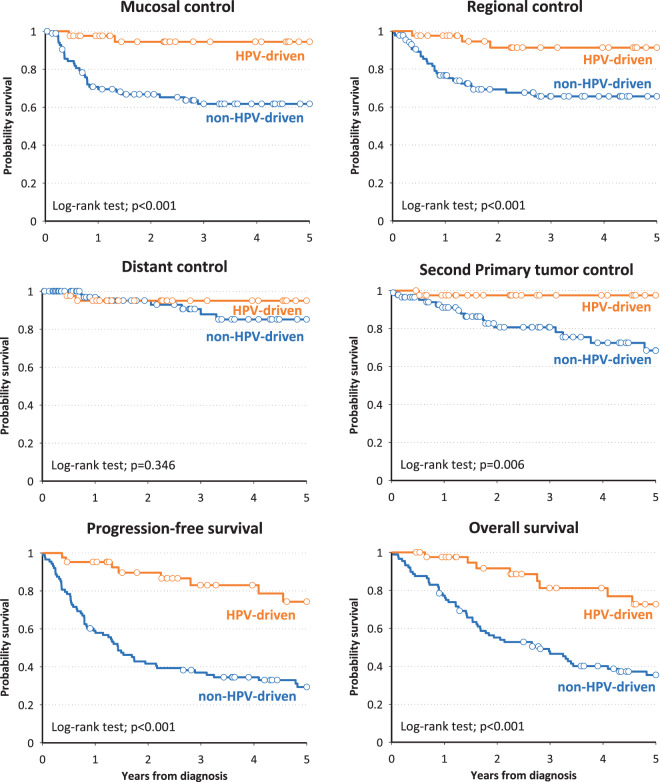
Kaplan Meier estimates of survival outcomes in HPV-driven and non-HPV-driven oropharyngeal cancers. Distinct panels show different clinical outcomes.

Different socio-demographic and clinical characteristics were evaluated and correlated with clinical outcome (Table 3). TNM (Tumor Nodes Metastases) stage as defined in the 8th edition was statistically correlated with progression-free (PFS) and overall (OS) survival; PFS hazard ratios (HR) for stages III and IV were 2.82 and 4.65, respectively; the corresponding HRs for OS were 2.62 and 3.53, respectively. A significant correlation was also found between current alcohol drinking and both PFS and OS (HR 2.59 and 3.18, respectively). Among HPV-driven cases, a significantly better control of mucosal (p = 0.003) and regional (p = 0.006) relapse and of second primary tumor (p = 0.009) was recorded, whereas a not significant difference (p = 0.360) for distant control was observed (Table 4); PFS and OS were highly significantly (p < 0.001) improved (Fig. 3). Four out of five patients with p16 positive/HPV-DNA negative tumors showed an unfavorable outcome (Supplementary Table 2).

**Table 3:** Hazard ratio (HR) and corresponding 95% confidence interval (CI)^a^ for progression-free survival and overall survival according to socio-demographic and clinical characteristics.

	Pts	Progression-free survival^b^				Overall survival			
		n	(%)	HR	(95% CI)	n	(%)	HR	(95% CI)
**Gender**									
Female	35	16	(45.7)		Ref.	15	(42.9)		Ref.
Male	95	63	(66.3)	0.86	(0.47–1.56)	60	(63.2)	0.76	(0.41–1.41)
**Age (years)**									
<65	68	42	(61.8)		Ref.	39	(57.4)		Ref.
≥65	62	37	(59.7)	1.33	(0.83–2.11)	36	(58.1)	1.60	(0.99–2.59)
**Year of diagnosis**									
2000–2006	36	28	(77.8)		Ref	28	(77.8)		Ref
2007–2012	42	33	(78.6)	1.84	(1.05–3.23)	30	(71.4)	1.41	(0.80–2.49)
2013–2018	52	18	(34.6)	0.83	(0.44–1.55)	17	(32.7)	0.90	(0.47–1.72)
**Cancer subsite**									
Base of tongue	28	11	(39.3)		Ref.	10	(35.7)		Ref.
Tonsil	84	57	(67.9)	1.09	(0.53–2.24)	54	(64.3)	1.09	(0.52–2.27)
Other	18	11	(61.1)	1.02	(0.41–2.56)	11	(61.1)	1.15	(0.46–2.89)
**TNM stage (7th ed.)**^**c**^									
I-II	13	7	(53.9)		Ref.	7	(53.9)		Ref.
III	23	14	(60.9)	1.35	(0.53–3.42)	14	(60.9)	1.49	(0.59–3.80)
IV	94	58	(61.7)	1.89	(0.83–4.28)	54	(57.5)	1.81	(0.80–4.12)
**TNM stage (8th ed.)**									
I-II	38	12	(31.6)		Ref.	12	(31.6)		Ref.
III	27	17	(63.0)	2.82	(1.29–6.18)	16	(59.3)	2.62	(1.19–5.76)
IV	65	50	(76.9)	4.65	(2.27–9.49)	47	(72.3)	3.53	(1.76–7.09)
**Grading**									
G1-G2	63	48	(76.2)		Ref.	45	(71.4)		Ref.
G3	55	28	(50.9)	0.57	(0.57–0.95)	27	(49.1)	0.79	(0.47–1.34)
**Tobacco smoking**									
Never	26	11	(42.3)		Ref.	10	(38.5)		Ref.
Former	30	19	(63.3)	1.11	(0.49–2.55)	18	(60.0)	0.99	(0.42–2.31)
Current	74	49	(66.2)	1.12	(0.52–2.41)	47	(63.5)	1.27	(0.58–2.79)
**Alcohol drinking**									
Never	54	18	(33.3)		Ref.	17	(31.5)		Ref.
Former	13	10	(76.9)	1.68	(0.75–3.80)	9	(69.2)	1.93	(0.82–4.54)
Current	61	50	(82.0)	2.59	(1.45–4.64)	48	(78.7)	3.18	(1.74–5.79)

^a^Adjusted for gender, age, year of diagnosis, TNM stage (8th ed.), and alcohol drinking. ^b^One patient was not evaluable for progression-free survival. ^c^Adjusted for gender, age, year of diagnosis, and alcohol drinking.

**Table 4:** Hazard ratio (HR) and corresponding 95% confidence interval (CI) for HPV-driven tumors vs non-HPV-driven tumors. SPT: second primary tumor

Survival outcome	Crude estimates^a^		Adjusted estimates^b^	
	HR	(95% CI)	HR	(95% CI)
Mucosal control	0.10	(0.02–0.44)	0.14	(0.03–0.61)
Regional control	0.18	(0.05–0.60)	0.28	(0.08–1.02)
Locoregional control	0.11	(0.03–0.36)	0.16	(0.05–0.52)
Distant control	0.66	(0.16–2.67)	1.64	(0.31–8.72)
SPT control^c^	0.09	(0.01–0.70)	0.10	(0.01–0.83)
Progression-free survival	0.22	(0.11–0.42)	0.27	(0.13–0.54)
Overall survival	0.25	(0.12–0.49)	0.33	(0.16–0.67)

^a^Adjusted for gender, age and year of diagnosis. ^b^Further adjusted for T status, N status, and alcohol drinking. ^c^Including only cancer of the head and neck, oesophagus and lung.

## Discussion

The incidence of HPV-driven OPC in North-Eastern Italy is lower than in Northern Europe and North America^7^, but the results of this study demonstrate that in this area it has significantly increased over the last two decades (raising from 17% in 2000–2006 to 46% in 2013–2018), thus reinforcing our previous observations^17^. Also in a previous Italian cancer registry-based study covering the period 1988–2012^18^, we showed a rising trend of HNSCC carcinomas potentially related to HPV (i.e. arising in the oropharynx). Altogether these observations indicate that a real increase of HPV-driven OPC cases is occurring in Italy. Moreover, our data highlight other two interesting aspects. First, the increase in HPV-driven cancers is more marked in females than males; second, HPV-driven OPC cases are no longer a disease restricted to the younger ages.

Differences by gender of HPV-driven OPC prevalence and time trends, with differential geographical distributions, have been already reported, and related to environmental risk factors and behavioural habits^2,13^. According to Combes *et al*.^19^, the male/female prevalence ratio we observed (0.49) is consistent with an excess of tobacco and alcohol related OPC in men compared to women in our geographic area. In Italy, both smoking and alcohol consumption are more prevalent among males than females: 25.5% versus 17% are current smokers, and 78.4% versus 46.1% are drinkers, respectively; moreover, alcohol drinkers are most frequent among current smokers (OR = 2.17), and alcohol consumption is more frequent in Northern than in Southern regions^20,21^. In relation to the temporal variations of HPV-driven OPC cases, an estimated higher prevalence in women than in men was reported and shown to be statistically significant in all European regions (except Northern Europe)^2^. In Italy, the age-adjusted incidence trends of SCC arising from HPV-related sites registered (10 cancer registries, for a total of 28 295 HNSCC cases) during the 1988–2012 period^18^ were stable in males and increased in women.

In line with recent reports^22^, we recorded HPV-driven cases with similar frequency in the different age groups. Indeed, while HPV-driven OPC was initially described as a new entity affecting mainly men aged 40 to 59 years^23^, in recent years it is becoming more common in older patients, and this change has been related to birth cohort effects^24^. The increase in the proportion of HPV-driven OPC cases among elderly patients bears the clinical challenge of treating patients with comorbidities for whom the survival advantage of HPV pathogenesis might be attenuated, and portend the need to include patients over 70 into the clinical trials assessing therapeutic and post-treatment surveillance protocols.

In comparison to non-HPV-driven cases, patients with HPV-driven OPC experienced significantly better survival outcomes, with the exception of distant control that was similar in the two groups. The HPV status of the tumor was established by the presence of HPV DNA sequences plus at least one additional marker, usually p16 overexpression, in order to identify truly HPV-driven tumors^15^. The presence of a single marker, i.e. HPV DNA positivity or p16 overexpression, does not always reflect a pathogenetic role of HPV. The detection of HPV-DNA and mRNA sequences or of HPV-DNA at high viral load has been shown to reliably identify transcriptionally active infections^15,25^. Moreover, since the HPV causality bears a prognostic significance, it would be important to define and standardize the laboratory protocol for the correct identification of HPV-driven OPC, also because many HPV-DNA detection assays, highly different in technical and analytical characteristics, are commercially available. Indeed, the clinical outcome reported for patients with discordant results for HPV-DNA and p16 is most often similar to non-HPV-driven cases^26,27^. The eighth edition of the AJCC/UICC (American Joint Committee on Cancer / Union for International Cancer Control) TNM staging system demonstrated a better prognostic capacity than the seventh edition for HPV-associated OPC, but confirms the concerns previously expressed^28^ on using p16 overexpression as a stand-alone test to define an OPC as HPV-driven. Indeed, in line with data of other studies^29^, in our study population the five patients with HPV-DNA-negative/p16-positive OPC showed an unfavorable clinical outcome, with local and regional, but not distant, disease progression (Supplementary Table 1), at variance with the observation by Rasmussen *et al*.^30^ of higher risk of M-site recurrence; the lower number of cases might explain this difference. Thus, we strongly recommend to use the double positivity for HPV-DNA/p16^INK4a^ to estimate the burden of HPV-driven OPSCC, obtain more precise prognostic information, and select patients in clinical trials exploring treatment de-intensification strategies for HPV-driven OPC.

HPV genotyping identified type 16 in 88% of cases, and types 33, 58 and 18 in the remaining 12%, a distribution similar to that recently reported in Eastern Denmark^14^. Interestingly, HPV33 was identified in 3 cases (7%), and non-16 types were found in older patients; similarly to what observed for cervical carcinoma, these observations confirm HPV33 as a highly oncogenic type^31^, and the incidence at younger age of HPV16-induced tumors^32^. Among the high-risk types, HPV16 is the most carcinogenic; in the cervix HPV16 infections are characterized by a much higher degree of developing high-grade disease within a shorter period of time than other high-risk types^32^. Whereas a poorer OS was observed in Norwegian patients with OPC associated with non-HPV16 types^33^, no differences in relation to HPV type emerged among our cases, but we analyzed a lower number of cases.

Strengths of our study are the use of two biomarkers for HPV-driven determination and the long period of patients’ enrollment and follow-up. A limitation is the relatively small number of patients included in our cohort.

In conclusion, we have observed an age-independent increasing prevalence of HPV-driven OPC over the last two decades also in North-East Italy. Patients with HPV-driven tumors defined by HPV-DNA positivity and additional markers (p16 and/or high viral load) showed an improved prognosis. Conversely, p16 positive/HPV-DNA negative patients had a worse outcome, underlying the importance to use additional markers to define the etiological role of HPV in these malignancies. Similar to cervical cancer, infections by non-HPV16 oncogenic types were restricted to older OPC cases. Systematic review and meta-analysis are required to investigate the prognostic impact of non-HPV16 oncogenic types in patients with OPC.

## Methods

### Aim, design and setting of the study

Aims of the study are to evaluate the temporal trend of HPV-driven OPCs in North-East Italy over a 18-year period, and to compare the clinical outcome of HPV-driven and chemically induced cases, also in relation to the HPV type. The OPC cases included in the present analysis represent a subset of a multicenter ongoing prospective cohort study on HPV involvement and prognostic biomarkers in head and neck squamous cell carcinoma (HNSCC) patients initiated in the year 2000. Patients were treated at 3 Ear, Nose, and Throat (ENT) Units located in North-East Italy (i.e., Treviso Regional Hospital, Hospital of Mirano, and Trieste Cattinara Hospital).

### Patients and tissue samples

All the available records of patients with OPC managed with curative intent from September 2000 to August 2018 were reviewed. Treatment planning was discussed by a multidisciplinary tumor board in order to decide the appropriate strategy, and management decisions were not guided by knowledge of HPV status. All patients underwent a regular follow-up until death or 31 July 2019, except one who at this date was still under treatment. Clinical, epidemiological, socio-demographic and behavioral data were also retrieved (gender, age, year of diagnosis, cancer subsite, TNM stage, histological grading when applicable (HPV-driven SCC is not graded), tobacco smoking, alcohol drinking). Histological diagnoses were determined by the local pathologists. All tumors were classified according to the WHO (World Health Organization) 4^th^ Edition and staged according to the AJCC 7^th^ Edition classification with reclassification also according to the 8^th^ Edition, that introduces a separate staging algorithm for HPV-driven OPC^34^.

The whole study was approved by the ethic committee for clinical experimentation (CEP) of Treviso (Ethic votes: 345/AULSS9 and 421/AULSS9). All patients signed an informed consent. Data on 63 patients have been included in previously published reports^17,35,36^. We confirm that all experiments were performed in accordance with relevant guidelines and regulations.

Fresh frozen (FF) and/or formalin-fixed paraffin-embedded (FFPE) specimens of the neoplastic lesions (primary tumor from all the enrolled patients, and recurrent tumors from HPV-driven cases) were collected.

DNA was extracted by the phenol-chloroform (PC9) method [as previously described by Baboci *et al*.^17^ or by the QIAamp DNA kit (Qiagen), according to the manufacturer’s instructions.

### HPV analyses

Search and typing of HPV DNA sequences were performed by PCR using MY09/MY11 primers and restriction fragment length polymorphism (RFLP) analysis of the amplified products, as previously described^17^. Quantitative real-time PCR (qPCR) was performed on HPV16-positive samples by using primers and probe specific for HPV16-E6, according to Peitsaro *et al*.^37^; two standard curves were obtained by amplification of 10-fold serial dilutions of full-length HPV16 genome (Clonit, Alfa Wassermann; 10^2^ to 10^6^ copies/µl) and the beta-globin gene (Roche; 2 to 200 ng). The E6 copy number and the amount of beta-globin were calculated; the viral load was expressed as HPV16 copies/cell.

### Immunohistochemistry for p16^INK4A^ protein expression

The expression of p16^INK4a^ protein (indicated as p16) was performed on FFPE sections by immunostaining using the primary antibody CINtec for V-kit (MTM laboratories, Heidelberg, Germany), as previously described^11^, or the BD Pharmingen IHC Detection kit, according to the manufacturer’s instructions. The expression results were scored as positive by using a 70% cut-point and considering nuclear and cytoplasmic stain distribution.

### Statistical analyses

A tumor was defined as HPV-driven by using at least two different biomarkers, usually HPV-DNA positivity and p16^INK4A^ overexpression; alternatively, positivity for HPV-DNA and a different biomarker (HPV16 mRNA, anti-E6/E7 antibodies, HPV16 high viral load, HPV16/18 E6 Oncoprotein) were used, as previously described^17^.

To facilitate statistical analysis due to the relatively small number of patients, clinical and socio-demographic data were divided into categories. HPV-driven and non-HPV-driven cancers were compared for each variable. Cochran-Mantel-Haenszel and Fisher exact tests were used, as appropriate. Similarly, median value of HPV16 copies for HPV16 positive cancers were compared for each variable, using Kruskall-Wallis test. For each patient, person-time at risk was calculated from diagnosis to event of interest or last available follow-up, whichever came first. Follow-up was truncated at 5 years. Event was defined as cancer recurrence at the UADT (Upper Aero Digestive Tract) site for mucosal control; neck lymph node failure for regional control; local cancer recurrence or regional failure for loco-regional control; distant metastasis for distant control; second primary tumor (SPT) for SPT survival; mucosal, regional or distant recurrence or death for progression-free survival (PFS); death for overall survival (OS). Survival probabilities were estimated according to Kaplan-Meier method and differences according to strata were tested through log-rank test. Progression-free survival and overall survival in the different categories identified by the clinical and socio-demographic data were evaluated by a multivariate model using Cox proportional hazards regression, adjusted for gender, age, year of diagnosis, TNM stage (8^th^ ed.) and alcohol drinking. A similar multivariate model was generated in order to investigate survival outcomes (mucosal control, regional control, loco-regional control, distant control, second primary tumors [SPT] control, progression-free survival, overall survival) in HPV-driven tumors vs. non-HPV-driven tumors, adjusting for gender, age and year of diagnosis to obtain crude estimates, and also for T status, N status and alcohol drinking for adjusted estimates.

### Ethics approval and consent to participate

The whole study was approved by the ethic committee for clinical experimentation (CEP) of Treviso (Ethic votes: 345/AULSS9 and 421/AULSS9). All patients signed an informed consent.

## Supplementary information

Supplementary Information.

## Data Availability

The data that support the findings of this study are available from the corresponding author upon reasonable request.
