# Supplementary material for: Age-independent increasing prevalence of Human Papillomavirus-driven oropharyngeal carcinomas in North-East Italy
Source: Sci Rep. 2020 Jun 9;10:9320. doi: 10.1038/s41598-020-66323-z (PMC7283341; doi:10.1038/s41598-020-66323-z)
Supplement: Supplementary file 1 — Supplementary Information. [file 41598_2020_66323_MOESM1_ESM.docx]

**Age-independent increasing prevalence of Human Papillomavirus-driven oropharyngeal carcinomas in North-East Italy with different distribution of viral types by age**

Annarosa Del Mistro^1*^, Helena Frayle^1^, Anna Menegaldo^2^, Niccolò Favaretto^3^, Silvia Gori^1^, Piero Nicolai^3^, Giacomo Spinato^2,4^, Salvatore Romeo^5^, Giancarlo Tirelli^6^, Maria Cristina da Mosto^2^, Jerry Polesel^7^, Paolo Boscolo Rizzo^2^

^1^Immunology and Diagnostic Molecular Oncology Unit, Veneto Institute of Oncology IOV – IRCCS, Via Gattamelata, 64, 35128 Padova, Italy – [annarosa.delmistro@iov.veneto.it](mailto:annarosa.delmistro@iov.veneto.it); [helena.frayle@iov.veneto.it](mailto:helena.frayle@iov.veneto.it); [silvia.gori@iov.veneto.it](mailto:silvia.gori@iov.veneto.it)

^2^Department of Neurosciences DNS, Section of Otolaryngology, University of Padova, P.le Ospedale 1, 31100 Treviso, Italy – [anna.menegaldo@hotmail.it](mailto:anna.menegaldo@hotmail.it); [giacomo.spinato@unipd.it](mailto:giacomo.spinato@unipd.it) ; [mariacristina.damosto@unipd.it](mailto:mariacristina.damosto@unipd.it); [paolo.boscolorizzo@unipd.it](mailto:paolo.boscolorizzo@unipd.it)

^3^Department of Neurosciences DNS, Section of Otolaryngology, University of Padova, Via Giustiniani 2, 35128 Padova, Italy - [niccolo.favaretto @unipd.it](mailto:niccolo.favaretto%20@unipd.it); [piero.nicolai@unipd.it](mailto:piero.nicolai@unipd.it)

^4^Department of Surgery, Oncology and Gastroenterology, Section of Oncology and Immunology, University of Padova, Via Gattamelata, 64, 35128 Padova, Italy - [giacomo.spinato@unipd.it](mailto:giacomo.spinato@unipd.it)

^5^Anatomical Pathology Unit, San Donà di Piave Hospital, Azienda ULSS 4 Veneto Orientale, Via Nazario Sauro 25, 30027 San Donà di Piave (VE), Italy - [salvatore.romeo@aulss4.veneto.it](mailto:salvatore.romeo@aulss4.veneto.it)

^6^Head and Neck Department, Cattinara Hospital, University of Trieste, Strada di Fiume 447, 34149 Trieste, Italy – [tirelli@units.it](mailto:tirelli@units.it)

^7^Unit of Cancer Epidemiology, Aviano National Cancer Institute, IRCCS, Via Franco Gallini, 2, 33081 Aviano (PN), Italy – [polesel@cro.it](mailto:polesel@cro.it)

*Corresponding author:

Annarosa Del Mistro, MD - Immunology and Diagnostic Molecular Oncology Unit, Veneto Institute of Oncology IOV – IRCCS, Via Gattamelata, 64, 35128 Padova, Italy

e-mail: [annarosa.delmistro@iov.veneto.it](mailto:annarosa.delmistro@iov.veneto.it) - ORCID ID: 0000-0001-7201-9374

**Supplementary Table 1.** Median HPV16 copies and interquartile range in 35^a^ HPV-driven oropharyngeal carcinomas according to socio-demographic and clinical characteristics.

|  | **Total**  **patients** | **HPV16 copies** | | **Kruskall-Wallis test** |
| --- | --- | --- | --- | --- |
|  |  | **Median** | **(Q1-Q3)** |  |
| Overall | 35 | 134.4 | (72.2-341.8) |  |
| Gender |  |  |  |  |
| Female | 16 | 95.2 | (33.8-243.3) |  |
| Male | 19 | 195.5 | (76.9-802.4) | p=0.112 |
| Age (years)^b^ |  |  |  |  |
| <65 | 19 | 94.5 | (37.9-196.8) |  |
| ≥65 | 16 | 283.2 | (83.7-680.2) | p=0.051 |
| Year of diagnosis |  |  |  |  |
| 2000-2006 | 5 | 558.0 | (256.1-806.7) |  |
| 2007-2012 | 11 | 90.1 | (76.9-341.8) |  |
| 2013-2018 | 19 | 112.6 | (18.5-318.6) | p=0.237 |
| Cancer subsite |  |  |  |  |
| Base of tongue | 8 | 213.1 | (96.3-287.3) |  |
| Tonsil | 27 | 95.9 | (69.4-370.1) | p=0.504 |
| T |  |  |  |  |
| 1-2 | 20 | 129.6 | (44.9-446.9) |  |
| 3-4 | 15 | 134.4 | (77.3-341.8) | p=0.739 |
| N |  |  |  |  |
| 0 | 7 | 335.9 | (76.9-877.0) |  |
| 1 | 7 | 134.4 | (18.5-256.1) |  |
| 2-3 | 21 | 94.5 | (69.4-318.6) | p=0.292 |
| TNM stage (8th ed.) |  |  |  |  |
| I-II | 22 | 171.1 | (76.9-335.9) |  |
| III -IV | 13 | 80.0 | (72.2-341.8) | p=0.562 |
| Grading |  |  |  |  |
| Well-moderately differentiated | 13 | 196.8 | (77.7-370.1) |  |
| Poorly differentiated | 17 | 94.5 | (51.9-318.6) | p=0.267 |
| Tobacco smoking |  |  |  |  |
| Never | 16 | 140.5 | (73.6-274.6) |  |
| Former | 8 | 407.0 | (74.5-804.6) |  |
| Current | 11 | 94.5 | (51.9-401.5) | p=0.472 |
| Alcohol drinking^c^ |  |  |  |  |
| Never | 24 | 115.1 | (53.7-327.2) |  |
| Current | 9 | 195.5 | (77.7-802.4) | p=0.476 |
|  |  |  |  |  |

^a^Data not available for seven patients; ^b^<65 years: 87.2 (28.2-196.3), ≥65 years: 283.2 (83.7-680.2); p=0.036; ^c^One former smoker excluded.

**Supplementary Table 2**. Sociodemographic and clinical characteristics of five patients with p16 positive oropharyngeal carcinoma with undetectable HPV DNA.

| **Sex** | **Age**  **(years)** | **Smoking**  **habits** | **Drinking**  **habit** | **Cancer site** | **TNM** | **Stage**  **7th ed.** | **Stage ^a^**  **8th ed.** | **Treatment** | **Treatment**  **response** | **Recurrence** | | **Survival** | |
| --- | --- | --- | --- | --- | --- | --- | --- | --- | --- | --- | --- | --- | --- |
|  |  |  |  |  |  |  |  |  |  | **Mo** | **Site** | **Mo** | **Vital status** |
| Man | 85 | Former | Current | BoT | T3 N2 | 3 | 2 | RT | RP | 0 | T | 0 | Dead |
| Man | 52 | Current | Current | Tonsil | T4a N2c | 4a | 3 | RCT | RP | 2 | T+N | 14 | Dead |
| Man | 61 | Current | Never | Tonsil | T4b N2b | 4b | 3 | RCT | PD | 1 | T+N | 6 | Dead |
| Man | 62 | Current | Current | Tonsil | T4a N2b | 4a | 3 | RCT | RC | --- | --- | 29 | Alive |
| Man | 61 | Current | Current | Tonsil | T4b N2c | 4b | 3 | RCT | RC | 9 | N | 33 | Dead |

^a^Using standard p16 positivity.
